# The Place and Prognostic Value of TERT Promoter Mutation in Molecular Classification in Grade II-III Glial Tumors and Primary Glioblastomas

**DOI:** 10.5146/tjpath.2021.01555

**Published:** 2022-05-19

**Authors:** Neslihan Kaya Terzi, İsmail Yılmaz, Aysim Buge Oz

**Affiliations:** Department of Pathology, University of Health Sciences Gaziosmanpasa Research and Training Hospital, Istanbul, Turkey; University of Health Sciences, Sultan 2. Abdulhamid Han Research and Training Hospital, Istanbul, Turkey; University of Cerrahpasa, Cerrahpasa Medical Faculty, Istanbul, Turkey

**Keywords:** Diffuse glioma, Primary glioblastoma, TERT p, Promoter mutation, Survival

## Abstract

*
Objective:* Diffuse gliomas, the most common primary malignant brain tumors, have been classified by the World Health Organization as class II-IV gliomas. After 2016, two mutations in the promoter region of the telomerase reverse transcriptase (*TERT*) gene were identified in addition to the IDH, 1p / 19q, and ATRX status.

*
Material and Method:* We identified 84 patients with grade II-IV glioma with IDH, ATRX, 1p / 19q and *TERT* status. All tumor samples were subjected to molecular genetic screening (Sanger sequencing for IDH and *TERT* mutations, fluorescence in situ hybridization for 1p/19q status) after histological diagnosis (immunohistochemistry for IDH1 R132H, ATRX, and p53) for a more precise molecular diagnosis. The confidence intervals were calculated at the 95% confidence level, and differences at p < 0.05 were considered statistically significant.

*
Results:* Primary glioblastomas had the highest frequency of *TERT* promoter mutations (25 of 28, 89.2%, p=0.006) followed by oligodendrogliomas (29 of 35, 82.8%, p<0.001) while astrocytomas showed the lowest frequency (3 of 15, 20%, p=0.107), and the positivity significantly differed among these three groups (p<0.001). *TERT* promoter mutations were more frequent in patients older than 55 years of age at diagnosis (p=0.023). The group with *TERT* promoter mutations, and without *IDH* mutations showed the worst overall survival. However, the presence of both *TERT* promoter and *IDH* mutations, which resembled oligodendroglial progression, showed best overall survival (p=0.042).

*
Conclusion:* The discovery of *TERT* promoter mutations in numerous gliomas has opened the door for a better molecular classification of gliomas, and *TERT* status is associated with survival. Further studies will help in elucidating the value of *TERT* promoter mutations as biomarkers in clinical practice, and eventual therapeutic targets.

## INTRODUCTION

Diffuse gliomas, the most common primary malignant brain tumors, have been classified by the World Health Organization (WHO) as class II-IV gliomas (1). After 2016, increasing and broad characterization of the genomic structure of gliomas has led to the identification of genetic and epigenetic markers useful for the molecular classification of these tumors (2). In addition to the IDH, 1p / 19q, and ATRX status, two mutations in the promoter region of the telomerase reverse transcriptase (*TERT*) gene were frequently identified (3). The mutation in the promoter region of this gene was first discovered in melanoma (4). In 2013, *TERT* promoter mutations were included in the molecular classification of gliomas (3). *TERT* is an important unit of the telomerase complex. It is known that upregulation of *TERT* increases telomerase activity, the basic survival ability of cancer cells, which allows for unlimited expansion of telomeres, and immortalization of cells (5). The mutation takes place in one of the two hot spots of the *TERT *gene, C228T and C250T, as C-T transition (6).

*TERT* mutations are usually investigated by sequencing or real-time PCR (7), and *TERT* promoter mutations occur in 70-80% of glioblastomas (GBM), 95% of oligodendrogliomas (OD), and 10-25% of astrocytomas (5). *TERT* promoter mutations are significantly inversely proportional to *IDH1 / 2* mutations (8). Changes in *TERT* and IDH are not only associated with specific histological glioma subgroups, but also are associated with a variable prognosis (9).

The literature on telomere-related mechanisms with glioma is rapidly increasing. Their prognostic and predictive roles are very interesting and may guide the clinical management of glioma patients. Yet, there are no studies investigating the distribution and significance of *TERT* promoter mutations in the WHO 2016 classification (10).

In the study conducted at Mayo Clinic, cases with 1 / 19q co-deletion, *IDH* mutation and *TERT* promoter mutation (triple-positive) were associated with an oligodendroglial phenotype and showed better overall survival (OS). Low-grade tumors (II and III) showing *TERT* and *IDH* mutations without 1p19q co-deletion tended to have a prognosis similar to triple-positive cases (11). However, patients without *TERT* and *IDH* mutations and those with 1p/19q co-deletion had very aggressive tumors and poor survival. Patients with wild-type (WT) *TERT*, IDH, and persevered 1p19q (triple negative) were associated with GBM, and had worse prognosis than triple-positive gliomas, but had a better prognosis than in patients with *TERT* mutations only (12).

## MATERIALS and METHODS

This study was approved by the medical ethics committee of the University of Health Sciences, Samatya Training Hospital (Approval No.: 06.07.2018 / 1337), and it was conducted according to the Declaration of Helsinki Principles.

### Study Population

We identified 84 patients (32 females and 52 males) with grade II-IV glioma with known IDH, ATRX, 1p / 19q and *TERT* status. Fifty-seven patients had WHO grade II-III tumors (24 oligodendrogliomas (OD), 14 anaplastic ODs, 13 astrocytomas, 5 anaplastic astrocytomas, and 1 anaplastic oligoastrocytoma with a low grade glioma component), and the remaining 27 samples represented high grade glioma (WHO grade IV/GBM) (Table 1).

**Table 1:** Clinicopathological variables of the 84 diffuse glioma patients.

**Demographics**	**n (%)**
**Gender**	
Male	52 (61.9)
Female	32 (38.1)
**Age (y), mean ± SD**	44.68±14.127
≤55	65 (77.3)
>55	19 (22.6)
**Tumor location**	
Frontal	48 (65.7)
Midline	16 (21.9)
Others	9 (12.3)
**WHO 2016 diagnosis**	
Diffuse astrocytoma, IDH-mutant	10 (11.9)
Diffuse astrocytoma, IDH-wildtype	3 (3.5)
Anaplastic astrocytoma, IDH-mutant	4 (4.7)
Anaplastic astrocytoma, IDH-wildtype	0 (0)
Oligodendroglioma, IDH-mutant & 1p/19q-codeleted	23 (27.3)
Anaplastic oligodendroglioma, IDH-mutant & 1p/19qcodeleted	14 (16.6)
Oligodendroglioma, NOS	1 (1.1)
Anaplastic oligoastrocytoma, IDH-mutant & 1p/19qcodeleted	1 (1.1)
Glioblastoma, IDH-mutant	0 (0)
Glioblastoma, IDH-wildtype	28 (33.3)
**Surgery**	
Biopsy only	10 (11.9)
Total resection	74 (88)
**Adjuvant treatment**	
None	4 (4.7)
Chemoradiation	80 (95.2)
**Survival outcome**	
Alive	61 (72.6)
Deceased	19 (22.6)

**SD: **Standard deviation

All tumor samples were subjected to molecular genetic screening after histological diagnosis for a more precise / molecular diagnosis (13,14). Tumors in the astrocytoma group were characterized by detectable immunoreactivity for IDH1 (R132H), p53 and loss of immunoreactivity for ATRX (15). All tumors in the OD group had *IDH1* mutation (R132H) and 1p / 19q co-deletion. GBMs were IDH wild type (IDH-WT) (1).

### Immunohistochemistry (IHC), Assessment of*TERT*promoter and IDH Mutation, Fluorescence in situ Hybridization (FISH)

Immunohistochemistry (IHC) was performed on at least one representative block using primary antibody against the following antigens- IDH1 R132H (Dianova, dilution 1:40), ATRX (Sigma, dilution 1:300), and p53 (Dako, dilution 1:50). Cases showing cytoplasmic positivity for IDH1 in tumor cells were considered positive. Loss of nuclear staining for ATRX in tumor cells (>90%) was considered positive for ATRX mutation. Nuclear positivity for p53 in >10% tumor cells was considered positive (16).

Mutations in the promoter region of *TERT* gene (chr5, 1,295,228C>T and 1,295,250C>T) and exon 4 of *IDH1* and *IDH2* genes (well-known hotspot regions for oncogenic mutations) were analyzed by PCR-based direct sequencing using representative formalin-fixed paraffin-embedded tumor samples. Tumor targets were manually microdissected from 5-μm thick unstained histological sections. After deparaffinization and rehydration, DNA was isolated from each target using QIAamp DNA FFPE Tissue Kit (50) (catalog #: 56404) (QIAGEN, Hilden, Germany) in accordance with the manufacturer’s instructions. DNA concentrations of samples were assessed spectrophotometrically using a Nanodrop 1000 spectrophotometer (Thermo Scientific, USA). PCR was performed in a Thermal Cycler (ABI, Applied Biosystems, USA) using HotStarTaq DNA Polymerase kit (catalog #: 203205) (QIAGEN, Hilden, Germany), and appropriate primers (*TERT* -Forward: 5’CAGCGCTGCCTGAAACTC’3, *TERT* -Reverse: 5’GTCCTGCCCCTTCACCTT’3, IDH1 exon 4-Forward: 5’ CCAAGTCACCAAGGATGCTG’3, IDH1 exon 4-Reverse: 5’ TCACATTACTGCCAACATGACTT’3, IDH2 exon 4-Forward: 5’ CCGTCTGGCTGTGTTGTTG’3, and IDH2 exon 4-Reverse: 5’ AGTCTGTCGCCTTGTACTGC’3).

PCR reactions were run as total volume of 50 µl reaction mixtures consisting of nuclease free water, 5 µl 10x PCR Buffer, 10 µl Q solution (for *TERT*), 1.5 µl 10 mM dNTP mix (ABI, Applied Biosystems, USA), 2 μl 25 mM MgCl2 (for *IDH1* and *IDH2*), 7 µl (for *TERT*) and 6 µl (for *IDH1* and *IDH2*) of each primer (4pmol/µl), 0.25 µl of Hot Start Taq DNA polymerase, and 50 ng of each tumor DNA. After an initial denaturation at 95°C for 15 minutes, 42 cycles were performed of 30 seconds denaturation at 95°C, 30 seconds annealing at 55°C (for *TERT*), and at 58°C (for *IDH1* and *IDH2*), and 45 seconds extension at 72°C, followed by a final extension of 10 minutes at 72°C. The intensity of the PCR products were checked by running 5 µl of each PCR reaction with 2 µl of loading dye on a 2% agarose gel. Reagent contamination control was achieved by examining the lane for “No DNA” blank tube. Then, all succeeded PCR products were purified using the QIAquick PCR Purification Kit (catalog #: 28106) (QIAGEN, Hilden, Germany) according to the manufacturer’s instructions. The purified amplicons were submitted to direct sequencing in both directions (forward and reverse) using reagents from the Big Dye Terminator v3.1 Cycle Sequencing kit (ABI, Applied Biosystems, USA) in accordance with the manufacturer’s protocol. After ethanol precipitation, subsequent products were run on the ABI-3730 (48 capillary) automatic sequencer (Applied Biosystems, USA). Bidirectional sequence traces were analyzed with SeqScape® Software v3.0 (Applied Biosystems, USA), and manually reviewed.

FISH analysis was performed on 5-micron-thick formalin fixed paraffin-embedded tissue samples. Deparaffinization, pre-hybridization and hybridization steps were conducted according to the datasheet. One hundred tumors cells were analyzed on the fluorescent microscope (Olympus BX61; Olympus Optical, Japan). The cells were captured on a computer system with a digital camera (XLMM, Dage-MTI, IN, USA), and compatible software (Duet®, Bioview Ltd., Israel). Dual-color paired probes for 1p and 1q (1p36 Spectrum Orange and 1q25 Spectrum Green, Vysis LSI probes, Abbott Molecular, Des Plaines, IL) were hybridized simultaneously on one slide, and similarly those for 19q, and 19p (19q13 Spectrum Orange and 19p13 Spectrum Green, Vysis LSI probes, Abbott Molecular, Des Plaines, IL) were used on a separate slide. A proportion is used due to the fact that a significant number of nuclei would have reduced comparison/control (green) signals because of tissue sectioning removing portions of the nuclei. Based on laboratory experience, a proportion <0.80 was considered as a deletion. The ratio of SpectrumOrange to SpectrumGreen signals (total orange/total green) was calculated.

### Statistical Analysis

Statistical analyses were performed using IBM SPSS Statistics for Windows, Version 21.0. (IBM Corp., Armonk, NY). Descriptive statistics were used to describe the data. Normal distribution was tested by the Kolmogorov-Smirnov and Shapiro-Wilk tests. Non-parametric data were compared using the Chi-square test. The Kaplan-Meier method was used for survival analysis, and the log-rank test (Mantel-Cox) was performed to compare the survival curves between the groups. The confidence intervals were calculated at the 95% confidence level and differences at p <0.05 were considered statistically significant.

## RESULTS

### Clinicopathologic Demographics

A total of 84 patients consisting of 52 men (61.9%) and 32 women (38.1%) were recruited. Their mean age was 44.68 ± 14.12 years (range 12–82 years). The mean duration of follow-up was 24-60 months. There were 65 patients (77.3%) aged less than 55 years, and 19 patients (22.6%) older than 55 years. The WHO 2016 revised diagnosis of the study group was as follows: 10 diffuse astrocytomas - IDH-mutant (11.9%); 3 diffuse astrocytomas- IDH-WT (3.5%); 4 anaplastic astrocytomas- IDH-mutant (4.7%); 23 ODs-IDH-mutant, and 1p/19q codeleted (27.3%); 14 anaplastic ODs, IDH-mutant & 1p/19q-codeleted (16.6%); 1 OD NOS (1.1%); 1 anaplastic oligoastrocytoma (1.1%), and 28 GBM-IDH-WT (33.3%). Biopsy was carried out only in 10 patients (11.9%), and debulking surgery was performed in 74 patients (88%). 80 patients (95.2%) were treated with some form of adjuvant radiotherapy and/or chemotherapy, while 4 (4.7%) patients did not receive any adjuvant radio-chemotherapy. 61 patients were alive (72.6%) while 19 patients (22.6%) died (Table 1).

### *TERT*Promoter Mutations and Alterations in Other Genes

**Figure 1:**
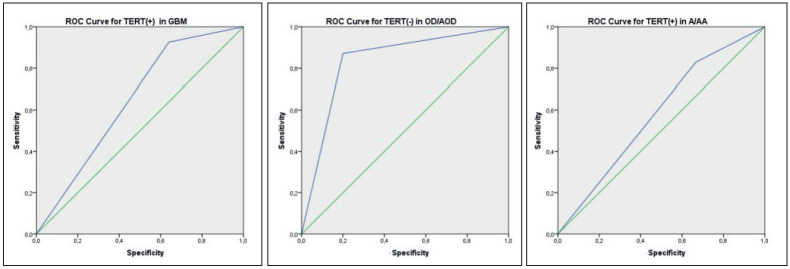
Specificity and sensitivity of TERT mutation in glioblastoma, oligodendroglioma / anaplastic oligodendroglioma, and astrocytoma / anaplastic astrocytoma.

Overall, *TERT* promoter mutations were present in 57 of 84 (67.9%) gliomas [20 (60.6%) in grade II, 12 (66.6%) in grade III, and 25 (92.5%) in grade IV]. The C228T mutation was detected in 42 tumors (53.8%), whereas the C250T mutation was found in 15 cases (19.2%), supporting the predominance of the C228T mutation in glioma. Primary glioblastomas had the highest frequency of *TERT* promoter mutations (25/28; 89.2%, p=0.006) followed by oligodendrogliomas (29/35; 82.8%, p<0.001), while astrocytomas showed the lowest frequency, with 3 out of 15 (20%, p=0.107) samples showing mutations (Table 2). *TERT* promoter mutations were found to be associated with high grade (grade III/IV) tumors when compared to lower grade (grade II) lesions (p<0.033), and were more frequent in patients older than 55 years of age (p=0.023). A statistically significant association was observed between the *TERT* mutation and the diagnosis (p<0.001). We performed post hoc power analysis by G*Power 3.1.9.2. The Type II error (β) probability was less than 0.01 (power >0.99) for the combined diagnostic algorithm (Figure 1).

**Table 2:** The frequency of *TERTp* mutations.

**Diagnosis **	**C228T mutation**	**C250T mutation**	**Both** ***TERT*** **mutations**
	**n (%)**	**n (%)**	**n (%)**
OD, *IDH* mutant	18/23 (78.2)	5/23 (21.7)	23/26 (88.4)
OD, *IDH* wildtype	1/2 (50)	1/2 (50)	2/4 (50)
A-AA, *IDH* mutant	1/2 (50)	1/2 (50)	2/12 (16.6)
A, *IDH* wildtype	1/1 (100)	0 (0)	1/2 (50)
GBM, *IDH* wildtype	19/25 (76)	6/25 (24)	25/28 (89.2)

**OD:** Oligodendroglioma, **A:** Astrocytoma, **AA:** Anaplastic astrocytoma, **GBM:** Glioblastoma.

*IDH* gene mutations were present in 44 tumors (42 of these being *IDH1* R132H mutations). Twenty-nine of these (corresponding to oligodendrogliomas) harbored a concomitant 1p/19q co-deletion, and all of them except three were also *TERT* promoter mutant.

**Figure 2:**
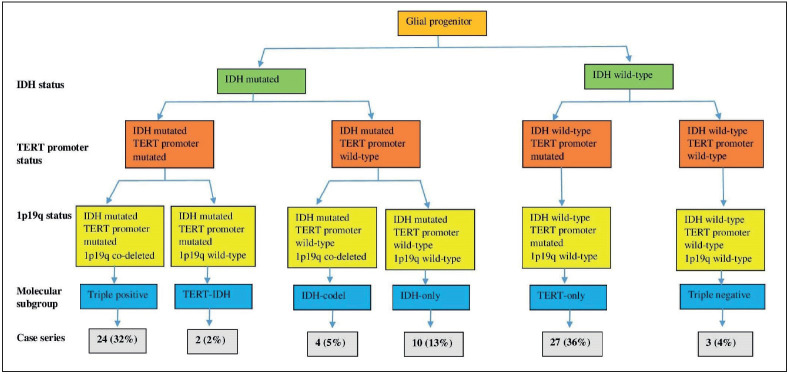
Molecular classification of diffuse glioma and frequency of each subgroup in our study.

The *TERT* p mutation and *IDH* mutation were more highly associated compared to *TERT* p with IDH-WTs (P<0.001 and p=0.655, respectively). However, in oligodendroglial tumors, *TERT* promoter and *IDH *mutations occurred together (p<0.001). 24 of 26 IDH/*TERT* double mutant tumors had the 1p/19q co-deletion. In primary glioblastomas, there were no *IDH* mutations, as expected. In astrocytomas, *TERT* promoter and *IDH* mutations were both found in only 2 of 15 cases (Figure 2).

Moreover, *TERTp* and *TP53* mutations in low-grade and anaplastic gliomas were mutually exclusive, where none and 5.5% of *TERT* mutated tumors harbored *TP53* mutations, respectively. A statistically significant association was observed between the *TERT* mutation and *p53* mutation (p<0.001).

*TERT* promoter mutations were mutually exclusive with ATRX deficiency. However, no statistically significant association was observed between the *TERT* mutation and ATRX mutation (p=0.533).

### Effect of*TERT*Promoter Mutations on Survival

**Figure 3:**
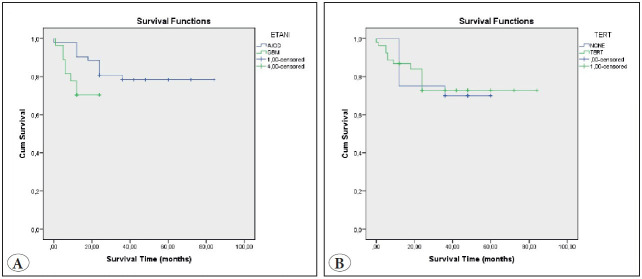
Survival times of anaplastic oligodendroglioma and glioblastoma cases **(A)**. Survival times of cases with TERT mutation **(B)**.

Results of the Kaplan-Meier survival analysis are shown in Figure 3A,B.

The overall cumulative survival in cases with *TERT* mutation was 64.94 months (95% CI=55.58-74.31) and overall cumulative survival in cases without *TERT* mutation was 46.80 months (95% CI=37.71-55.89) (p=0.870) (Figure 3B). Patients carrying the C250T mutation had slightly longer survival compared to patients with the C228T mutation (78.5% and 76.9%).

Moreover, the *TERT* mutation frequency, both C228T and C250T, increased with age: <40 years: 65.5% (19/29); 40-55 years: 66.6% (20/30), and >55 years: 94.7% (18/19). Compared to patients with WT-* TERT*, younger patients with *TERT* mutation survived longer, but patients with *TERT* mutation who were aged >55 years had shorter survival.

Stratification of the patients based on the mutational status of *TERT* promoter and *IDH *resulted in three groups with different overall survival. The group with *TERT* promoter mutations and no IDH mutations had the worst overall survival (median survival 12.29 months), the 2nd worst overall survival rate was the group without *TERT* and IDH mutations (median survival 24 months). Best overall survival was associated with the presence of both *TERT* promoter and *IDH* mutations (median survival 38.07 months), which resembles oligodendroglial progression (p=0.042).

Survival analysis in patients with primary glioblastomas did not reveal any effect of the *TERT* promoter mutations. Both patients with and without *TERT* promoter mutations had a median survival of 12 months (Figure 3A).

In diffuse and anaplastic gliomas that showed *TERT* promoter mutations were associated with poor survival while 1p/19q co-deletions had a favorable effect. Survival in gliomas with a *TERT* mutation and 1p19q co-deletion (median survival 58.2 months) was higher than in those without *TERT* mutation and 1p19q codeletion (median survival 42 months).

## DISCUSSION

Gliomas are the most common and aggressive primary brain tumors in adults (2). For diffuse gliomas, *IDH* mutations and 1p/19q co-deletion constitute the main components of the integrated WHO 2016 diagnosis. Molecular classifications proposed in the literature include the combination of the IDH mutations, the 1 p / 19q co-deletion, and the telomere maintenance mechanism as defined by alterations in *TERT* (15). To determine whether the *TERT* status provides additional prognostic information, we analyzed the relationship between overall survival in glioma patients classified according to the WHO 2016 criteria in our cohort.

Recent studies have shown that this classification has certain limitations for precise prediction of clinical outcomes and strategies for gene target therapy (16). Therefore, a more objective glioma classification is needed to guide diagnosis and treatment strategies.

Telomeres are structures at the end of eukaryotic chromosomes and are responsible for the deterioration of chromosomes, end-to-end fusion, and protection from rearrangement (17). Telomerase maintains the appropriate telomere length by adding repeated telomeric sequences to the 3’ ends of the telomeres. Abnormal telomerase activity plays a role in the initiation and development of cancer and other diseases related to aging (18). *TERT* is overexpressed in many human cancers (19). *TERT* promoter mutations were first found in melanoma and were thought to represent the tumorigenic mechanism (16).

The discovery of *TERT* p mutations in numerous gliomas has opened the door for a better molecular classification of gliomas in 2013 (20,21). *TERT* promoter mutations occur in 70-80% of glioblastomas, 95% of oligodendrogliomas, and 10-25% of astrocytomas (5,7,11).

The prevalence of *TERT* promoter mutations associated with various histological categories was similar to other studies (22,23). As expected, IDH-mutant tumors were prevalent predominantly in younger adults, while all IDH-WT/ *TERT* -mutant tumors apart from a single pediatric tumor occurred in adults. Our findings are consistent with the concept that IDH-WT/ *TERT*-mutant diffuse gliomas represent a clinicopathological part of primary glioblastomas and their precursors (24,25). In any case, inclusion of *TERT* promoter mutation analysis into a diagnostic molecular panel shifts the vast majority of otherwise marker-negative diffuse gliomas into a biologically plausible category. Furthermore, *TERT* promoter mutation status is of prognostic significance not only in diffuse gliomas with non-glioblastoma histology (11,25), but also in glioblastoma (10,26). The present study demonstrates that adding the *TERT* promoter mutation status to standard markers, as suggested in the literature, results in a precise molecularly directed classification with IDH-WT/* TERT* -mutant tumors representing the primary glioblastoma group (27).

Age was another parameter for survival. Patients aged >55 years who had *TERT* mutation had worse survival compared to patients without the mutation.

In the whole cohort, patients with C250T mutations tended to have longer survival compared to patients with C228T mutations. We found that occurrence of the C228T and C250T mutations were mutually exclusive in our cohort, and that C228T (84.2%) was more common than C250T (15.8%).

With rare exceptions, *TERT* promoter mutations (8), which lead to an increase in telomerase expression, and inactivating mutations in the thalassemia/mental retardation syndrome X-linked (ATRX) gene, are mutually exclusive, and are associated with different molecular tumor subclasses (28). Our study was the exception for mutual relationship of ATRX and *TERT* mutations, as we could not find any significant relationship (p=0.533). On the other hand, our study demonstrated that *TERT* promoter mutations have a significant inverse association with *P53* mutation (p<0.001), similar to previous studies (17).

*TERT* promoter mutations have been reported to be associated with aggressive behavior and poor outcome in various types of cancers (3). In this study, prognosis in glioblastoma cases with *TERT* mutation was worse than in oligodendrogliomas with *TERT* mutation. Cases with *TERT* mutation had a worse prognosis than cases without *TERT* mutation, but none of the relevant results in our study had statistical significance.

The combination of *TERT*p and *IDH* mutational status was a significant prognostic factor in grade II and III gliomas; this finding of a specific association between *IDH*/ *TERT*p group and low-grade glioma is consistent with results from a previous study (20,29). Eckel-Passow et al. (12) showed that patients with *IDH*-WT/*TERT*p-WT have poorer overall survival when compared with patients with *IDH*-mutated/*TERT*p or *IDH* mutation alone, but showed better overall survival when compared with patients with *IDH*-WT/*TERT*p-mutation (11). A recently published meta-analysis also suggested that combined *TERT*p-mutated/I*DH*-WT testing could act as a significant biomarker for poor prognosis in grade II and III gliomas (20).

Given that 67.9% of tumours being *TERT*p-mut, *TERT* is the most frequently mutated gene in gliomas thus far identified (5,8,30–32). Our data confirm the high frequency of *TERT*p-mutation in gliomas, and show that these mutations clusterise into specific entities, with distinct clinical significances.

In previous studies, the 1p/19q co-deletion was strongly associated with mutations in *TERT*p (33,34). In the present study, we found that 96.9% of patients with oligodendrogliomas had a *TERT*p mutation, whereas two patients with oligodendroglioma were *TERT*p-WT. On the contrary, there was a high frequency of *TERT*p mutation in cases with GBM that did not harbor the 1p/19q co-deletion, and therefore we conclude that the *TERT*p mutation is not exclusively associated with the 1p/19q co-deletion.

Regarding the *TERT*p mutation from a prognostic perspective in diffuse gliomas, previous studies have shown conflicting results. Labussiere et al. have found that *TERT*p mutations may be associated with poorer outcome in high-grade gliomas (31), however, Pekmezci et al. have reported that *TERT*-mutants had significantly worse survival only in IDH-WT astrocytoma, which includes grades II and III (10). Such contradictory effects of *TERT*p mutation on patient outcome between groups have been reported previously (11). Aibaidula et al. found comparable results to those of Pekmezci et al., and concluded that adult IDH-WT lower-grade gliomas should be further classified by *TERT*p mutation status (35).

According to Cimpact now update 3, IDH wild type diffuse or anaplastic astrocytomas will have a worse prognosis as grade IV glioblastomas. The following molecular methods can be used to distinguish these cases: EGFR amplification, losses of chromosome 10 (whole chromosome, 10p or 10q), gains of chromosome 7 (whole chromosome, 7p or 7q), *TERT* promoter mutations, homozygous deletion of CDKN2A/B, and large-scale, microarray based DNA methylation profiling. According to the results of the current study, histologic IDH-wildtype diffuse astrocytic gliomas of WHO grade II or III that carry EGFR amplification, +7/−10 or *TERT* promoter mutation are associated with significantly shorter survival compared to patients with other WHO grade II or III gliomas, and outcomes are similar to those in patients with IDH-wildtype glioblastoma (27). Molecular studies other than *TERT* promoter mutation could not be performed, and this is a limitation of our study. We aim to fill this gap in future studies.

*IDH* mutation was detected in 16 cases with midline localization in our study and their grade was II. In these cases, which are known to have a good prognosis in follow-up, diffuse midline glioma was evaluated by considering it in the differential diagnosis. However, *H3F3A K27M* mutation could not be detected with immunohistochemical or molecular methods.

*TERT* promoter mutations were shown to have inverse prognostic effects in IDH-mut and IDH-WT WHO grade II/III gliomas. Our study strongly supports using *TERT* and IDH genotyping in WHO grade II/III gliomas as a reliable and reasonable test that can help clinicians predict patient outcome more precisely than using only conventional histology, or *TERT* or IDH status alone. It is important to note that gliomas with concurrent *TERT* promoter and *IDH *mutations are almost always accompanied with 1p19q co-deletion, which is the hallmark of oligodendroglioma according to the WHO classification 2016, and it can help to explain why gliomas with coexisting *IDH* and *TERT* promoter mutations are most likely associated with favorable outcome.

Data suggest that patients with *TERT* promoter mutations in tumors probably require more aggressive treatment than their WT counterparts. Further studies will help elucidating the value of *TERT* promoter mutations as biomarkers in clinical practice and eventual therapeutic targets. Expression data and an association with shorter telomeres already strongly indicate the role of the *TERT* promoter mutations not only in glioma, but in many other cancer types, and future functional studies will aid in placing the *TERT* promoter mutations into the right context.

## Conflict of Interest

The authors declare that they have no conflict of interest.

## Funding

This study was supported by the University of Health Sciences Scientific Research Unit (Project number 2018/064).
